# COVID-19 and trained immunity: the inflammatory burden of long covid

**DOI:** 10.3389/fimmu.2023.1294959

**Published:** 2023-11-28

**Authors:** Jienan Gu, Qianhui Liu, Jiale Zhang, Shijie Xu

**Affiliations:** ^1^ Institute of Basic Theory for Chinese Medicine, China Academy of Chinese Medical Sciences, Beijing, China; ^2^ The First School of Clinical Medicine, Zhejiang Chinese Medical University, Hangzhou, China

**Keywords:** post-acute sequelae of COVID-19 symptoms, monocytes, innate immune, hematopoietic stem and progenitor cells, interleukin-6, interleukin-1β, chronic inflammation

## Abstract

Severe COVID-19 elicits excessive inflammation mediated by innate immune cells like monocytes. Recent evidence reveals extensive epigenetic changes in monocytes during recovery from severe COVID-19, including increased chromatin accessibility at genes related to cytokine production and leukocyte activation. These changes likely originate from the reprogramming of upstream hematopoietic stem and progenitor cells (HSPCs) and represent “trained immunity”. HSPC-to-monocyte transmission of epigenetic memory may explain the persistence of these monocyte alterations despite their short lifespan. IL-6 appears pivotal for imprinting durable epigenetic modifications in monocytes during acute infection, with IL-1β potentially playing a contributory role. The poised inflammatory phenotype of monocytes post-COVID-19 may drive chronic inflammation and tissue damage, contributing to post-acute sequelae of COVID-19 symptoms. COVID-19 could also exacerbate inflammation-related diseases, such multisystem inflammatory syndromes, by altering innate immune tendencies via hematopoietic epigenetic reprogramming. Further clinical investigations quantifying inflammatory mediators and mapping epigenetic changes in HSPCs/monocytes of recovering patients are warranted. Research should also examine whether COVID-19 elicits transgenerational inheritance of epigenetic alterations. Elucidating mechanisms underlying COVID-19-induced monocyte reprogramming and developing interventions targeting key inflammatory regulators like IL-6 may mitigate the sustained inflammatory burden imposed by the aberrant trained immunity post-COVID-19.

## 1 Introduction

Severe COVID-19 involves excessive inflammation driven by the innate immune system, including monocytes and macrophages (1). Monocytes produce a significant amount of inflammatory cytokines during SARS-CoV-2 infection (2). Studies have shown an increased presence of classical monocytes expressing inflammatory genes in the blood and lungs of COVID-19 patients (3). Activation of monocyte-derived macrophages contributes to cytokine storms (1, 4). Many patients experience post-acute sequelae of SARS-CoV-2 infection (PASC) or long COVID following initial recovery (5, 6). Symptoms of long COVID include persistent fatigue, dyspnea, and more lasting for up to 12 weeks (7). Elevated levels of inflammation biomarkers indicate sustained inflammation in various organs (8). This may be related to the concept of “trained immunity,” where immune cells exhibit enhanced responsiveness following infection (9, 10).

Multiple studies have indicated that monocytes exhibit trained immunity after infection and vaccination (11–13). Influenza vaccination induces dynamic chromatin remodeling in monocytes (14). BCG vaccine alters immune and metabolic genes in monocytes (15). These findings suggest that acute inflammation can lead to trained immunity and functional changes in monocytes. Epigenetic changes in monocytes during the recovery phase of COVID-19 suggest acquired trained immunity (16–18). These changes can persist for several months to a year (17). Monocytes from recovered patients exhibit enhanced responsiveness upon stimulation (17). Similar monocyte epigenetic changes have been observed after other inflammatory stimuli, including influenza vaccination and LPS (10, 19). The poised chromatin state at inflammatory genes renders monocytes hyperresponsive to stimuli, resulting in exacerbated inflammation (12, 15).

Based on these findings, we propose that the observed epigenetic changes in monocytes during the recovery process after severe COVID-19 represent a form of “trained immunity” (20). This epigenetic reprogramming likely originates in hematopoietic stem and progenitor cells (HSPCs) and is conveyed to monocyte progeny, persisting for months due to the longevity of HSPCs (21). Changes in HSPCs and monocytes may result in sustained cytokine release and inflammation-mediated tissue damage, contributing to the persistence of PASC and imposing an inflammatory burden. Understanding the biological basis of this inflammatory burden and assessing its implications is crucial for guiding public health decisions and future research.

## 2 Trained immunity in COVID-19

### 2.1 Trained immunity in monocytes following COVID-19

#### 2.1.1 Proinflammatory tendencies

Recent studies have discovered that circulating CD14+ monocytes from COVID-19 convalescent patients exhibit significant changes in chromatin accessibility through ATAC-seq analysis (17). Cheong et al. performed bulk and single-cell ATAC-seq on monocytes from COVID-19 patients 4-12 months after infection and found many regions related to monocyte activation and cytokine production, such as TNIP2, MAPKAPK2, IL21R, MMP1 and CREB1, remained more accessible compared to healthy controls (17). Single-cell RNA sequencing analysis also showed that monocytes from COVID-19 recovering patients have persistent transcriptomic changes, activating expression of genes related to antiviral and inflammatory responses (17). Functional experiments confirmed that monocytes from COVID-19 convalescents have enhanced cytokine release in response to Toll-like receptor agonist R848 and interferon-α (17). These epigenetic changes were evident in both classical CD14+ and non-classical CD16+ monocyte subsets (22). These changes represent a trained immunity phenotype. Similarly, You et al. found persistent chromatin remodeling in recovered COVID-19 patients’ monocytes using single cell multi-omics, with accessibility changes annotated to viral response, leukocyte migration, and maturation programs (22). Together, these studies demonstrate that monocytes undergo epigenetic reprogramming after COVID-19 infection, resulting in durable innate immune memory effects with proinflammatory tendencies, despite the short lifespan of these cells.

#### 2.1.2 Immune metabolic reprogramming

Both *in vitro* and *in vivo* studies have indicated that metabolic reprogramming is a critical event in inducing trained immunity, which is associated with enhanced protection against unrelated pathogens (15). Relevant research has observed changes in the metabolism of monocytes following COVID-19 vaccination (23). In individuals who received a single dose of the ChAdOx1 nCoV-19 vaccine, monocytes exhibited enhanced glycolytic capacity after two months (23). Furthermore, on day 56 after vaccination, these metabolically reprogrammed monocytes produced increased levels of the pro-inflammatory cytokine IL-1β, indicating an enhanced functional output (23). These findings may be relevant to the trained immunity and inflammatory response elicited by COVID-19 infection.

#### 2.1.3 Reprogramming of HSPCs

**Figure 1:**
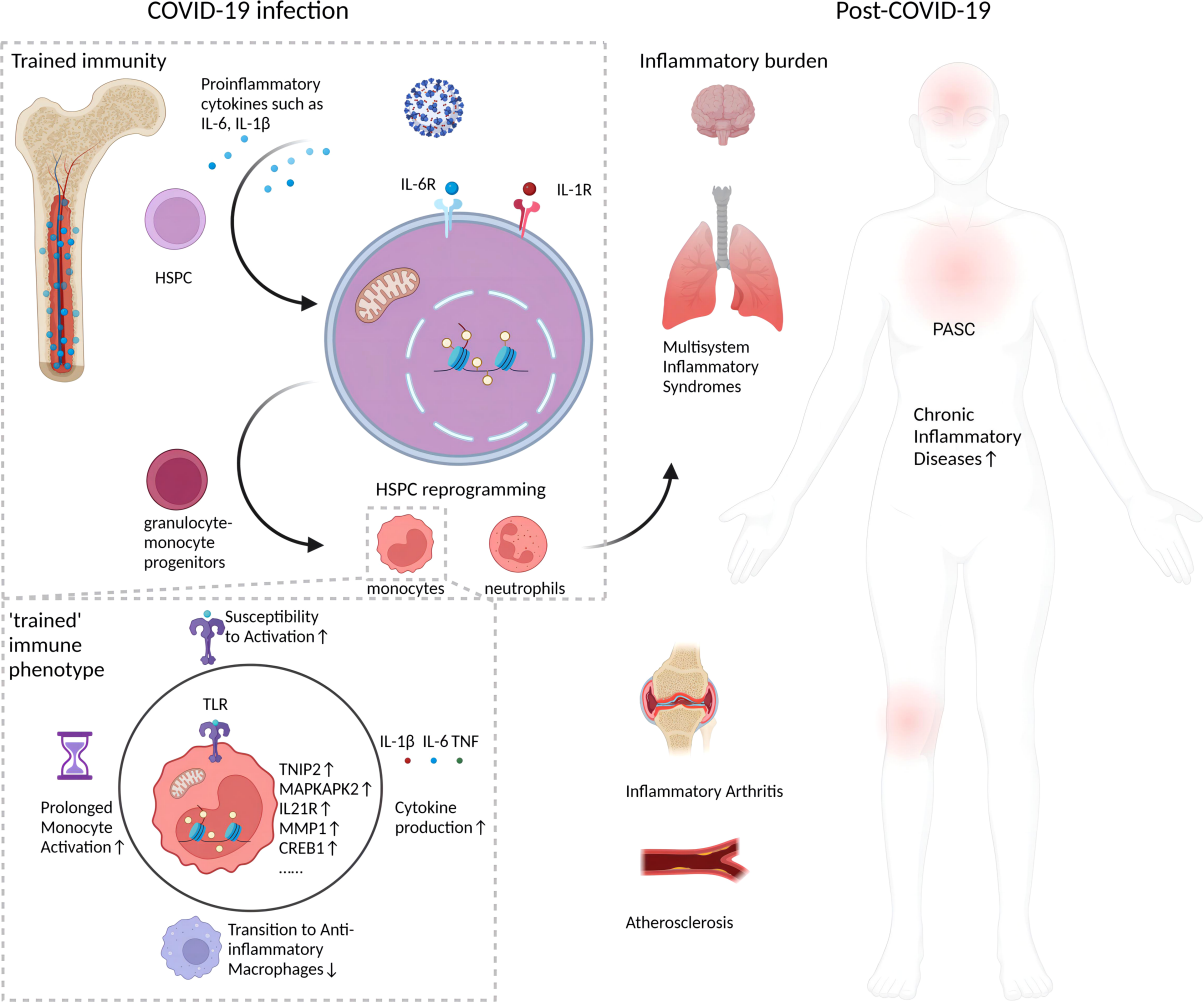
Long-term impact of epigenetic reprogramming in monocytes after COVID-19 infection. Following resolution of SARS-CoV-2 infection, monocytes display enduring epigenetic modifications that sustain a “trained” immune phenotype. This is characterized by enhanced chromatin accessibility, stable transcriptomic shifts, and metabolic reprogramming. Such alterations engender an augmented responsiveness to stimuli, precipitating a continuous secretion of pro-inflammatory cytokines, for instance, IL-1β. This sustained cytokine production could potentially intensify the pathophysiology of inflammatory disorders. The inception of this epigenetic memory is postulated to stem from initial reprogramming events in HSPCs, which subsequently imprint a lasting, altered functionality on monocyte lineages. This may manifest as persistent, subclinical inflammation and incremental tissue damage, factors implicated in the pathogenesis of post-acute sequelae of SARS-CoV-2 infection (PASC), or long COVID. Furthermore, the figure underscores the inhibitory effects of epigenetic reprogramming on the physiological transition of monocytes/macrophages from an inflammatory to an anti-inflammatory, reparative phenotype, which may impede tissue regeneration. Collectively, these phenomena establish a protracted inflammatory milieu with potential long-term health ramifications for individuals beyond the acute phase of COVID-19.

The persistence of monocyte epigenetic changes months to a year after COVID-19 recovery implies a form of innate immune memory. Classical monocytes normally circulate for only a few days before differentiating into macrophages (24). Thus, the prolonged presence of altered chromatin accessibility patterns in COVID-19-recovered patients’ monocytes suggests the changes originated in upstream HSPCs (17). HSPCs giving rise to monocytes can survive for years, allowing them to maintain epigenetic memory of prior immune activation that shapes the phenotypes of their differentiated progeny (25). Evidence indicates HSPCs acquire durable epigenetic remodeling after inflammatory challenges that alter myeloid cell development (11, 25). Similar forms of monocyte innate memory have been observed after viral infections and vaccinations *in vitro* (26). The longevity of these monocyte epigenetic marks implies an ancestral imprinting event in hematopoietic progenitors. Further integrated analyses of isolated HSPCs and monocytes in recovering COVID-19 patients will help confirm this. Hematopoietic stem cell epigenetic remodeling has also been reported in response to inflammatory stimuli like lipopolysaccharide (LPS), with changes enriched at genes involved in immune response (27). The enduring changes in HSPCs and monocytes behavior post-COVID-19, as depicted in **Figure 1**, suggest a “trained immunity” with lasting epigenetic reprogramming. These changes, persisting well after acute infection, may fuel the chronic inflammation seen in PASC.

### 2.2 Trained immunity in neutrophils following COVID-19

Patients with PASC exhibit characteristics of trained immunity in their neutrophils, specifically in a subset called low-density neutrophils (LDNs). The LDNs, which are characterized by inflammation and metabolic reprogramming, were studied using single-cell RNA sequencing technology. The study identified distinct subsets of LDNs in recovering Omicron patients, including MME^hi^, MX1+, and PI3+ LDNs (28). These subsets demonstrated elevated levels of inflammatory cytokines and chemokines, similar to EGR1+ monocytes. MX1+ LDNs exhibited a strong response to interferons. On the other hand, CEACAM8+ LDNs displayed superior activity in innate immune defense, including antimicrobial peptide production, neutrophil granule dissolution, and involvement in tissue remodeling processes (28). Another significant difference between the subsets was observed in their metabolic pathways. MME^hi^, MX1+, and PI3+ LDNs primarily exhibited characteristics of nicotinamide salvage metabolism, while CEACAM8+ LDNs predominantly showed features of glycogenolysis, mTOR, and pentose phosphate pathways (28). This further suggests a close association between immune function and metabolic pathways. These findings indicate that LDNs in patients with post-acute COVID-19 sequelae exhibit trained immunity features, which may result in prolonged inflammatory burden.

## 3 Potential immunity trained under COVID-19

### 3.1 IL-6 is an important cytokine for upstream epigenetic alterations in monocytes

IL-6 is a pleiotropic cytokine that has emerged as a key driver of inflammatory responses and disease severity in COVID-19. Serum IL-6 levels are markedly elevated in patients with severe COVID-19 and predictive of mortality (29, 30). Sources of excessive IL-6 likely include activated monocytes/macrophages and endothelial cells (31). IL-6 can activate monocytes and macrophages, leading to the release of downstream inflammatory mediators (32). Acute SARS-CoV-2 infection induces emergency hematopoiesis and biased myelopoiesis (33, 34), but the contribution of early IL-6 activity to these long-term hematopoietic changes is unknown. In other inflammatory contexts, IL-6 instructs myeloid skewing of HSPCs, altering differentiation trajectories of monocytes and tissue macrophages (35).

Emerging evidence indicates that blocking IL-6 signaling during acute COVID-19 can mitigate enduring epigenetic changes in monocytes. Cheong et al. found that patients treated with IL-6 receptor blockade during acute infection exhibited lower frequencies of altered progenitor and monocyte subpopulations months later compared to untreated patients (17). This included reduced biased myelopoiesis and fewer monocytes with hyperresponsive phenotypes. At the molecular level, IL-6R blockade prevented monocyte chromatin accessibility changes at loci linked to inflammation (17). These findings suggest that early IL-6R signaling contributes to the establishment of long-term monocyte epigenetic changes, rather than only transiently regulating inflammation. A murine coronavirus recovery model supports these observations. In this model, blocking the IL-6 receptor alleviated increased frequencies of enhanced myeloid progenitors and monocyte, as well as monocyte infiltration in the lungs and brain during the recovery phase (17). This animal model study corroborates the observations in humans, indicating that COVID-19 can induce an increase in the number and activity of monocytes in the peripheral tissues, and these effects can be attenuated by inhibiting the IL-6 pathway (17).

IL-6 signaling may impart epigenetic memory in monocytes and their progenitors through transcriptional mechanisms. The binding of IL-6 to its receptor activates downstream mediators like STAT3, which regulates target gene expression (36). Untreated COVID-19 patients exhibited increased persistent accessibility of STAT3 motifs in monocytes compared to the treated and healthy control groups (17). This implies early IL-6 activity mediates epigenetic memory, potentially through STAT3-dependent chromatin structural changes.

The epigenetic reprogramming of myeloid progenitors during infections may be conveyed to monocyte progeny, imprinting altered phenotypes. During hematopoiesis, epigenetic modifications are propagated from HSPCs through precursor stages to differentiated progeny via mitotic inheritance (37). In mouse models, trained immunity phenotypes can be adoptively transferred by transplanting reprogrammed HSPCs into recipients (38). Similarly, the altered monocyte phenotypes post-COVID-19 likely derive from epigenetic changes originally imprinted in HSPCs by early severe infection. The integrated study of isolated HSPCs and monocytes in the same recovering patients can help confirm progenitor-progeny epigenetic links (17). Understanding this process may inform strategies to reverse deleterious myeloid cell changes arising from progenitor reprogramming.

### 3.2 Speculating the role of IL-1β in trained immunity post-COVID-19 infection

IL-1β, a crucial pro-inflammatory cytokine, has gained considerable attention due to its potential involvement in the severity of COVID-19 (39, 40). Elevated serum levels of IL-1β have been observed in severe cases of COVID-19, and these increased levels are associated with the worsening of the disease (41). Additionally, IL-1β, along with IL-6 and TNF, has been associated with post-COVID-19 sequelae (42). Similar to IL-6, IL-1β plays a pivotal role in modulating trained immunity (43), but it has the potential to exacerbate inflammation, especially in cases of maladaptive trained immunity associated with various complications (44).

Beyond its role in inflammation, IL-1β also influences epigenetic changes and trained immunity, particularly in HSPCs and monocytes (44). Research indicates that IL-1β can promote epigenetic reprogramming in these cells, affecting their responses to subsequent stimuli. Notably, IL-1β affects metabolic reprogramming in HSPCs, driving them to rely more on glycolytic metabolism, leading to increased production of metabolic intermediates (45). These intermediates, in turn, enhance histone modifications such as lactylation, acetylation, and deacetylation, ultimately augmenting the production of pro-inflammatory cytokines like IL-6, TNF, and IL-1β (43). In specific pathological conditions, IL-1β has been identified as a key mediator of inflammatory diseases associated with trained immunity, such as periodontitis and arthritis (44). This suggests that IL-1β may contribute to exacerbated inflammatory responses observed in individuals with maladaptive trained immunity. The complex balance between IL-1β’s role in mediating immune responses and its potential for driving inflammation underscores its significance in trained immunity.

Although current research has not directly explored the specific role of IL-1β or other cytokines in trained immunity, it is hypothesized that IL-1β, possibly working in conjunction with IL-6 as an upstream cytokine, plays a role in inducing trained immunity. This hypothesis is based on IL-1β’s established role in other inflammatory responses unrelated to COVID-19 and its central role in regulating immune responses (44, 46, 47). Future studies should delve deeper into investigating the role of IL-1β in trained immunity following COVID-19. Such research may contribute to a better understanding of the molecular mechanisms underlying long-term sequelae of COVID-19 and offer potential guidance for the development of novel treatment strategies.

### 3.3 S1 protein and proinflammatory epigenetic changes in monocytes

In addition to cytokines, viral proteins themselves may induce proinflammatory epigenetic changes in monocytes (48). Studies have shown that the proportion of people with detectable circulating levels of the SARS-CoV-2 spike (S) protein S1 fragment is higher in populations with PASC (64%) compared to populations without PASC (35%) (49). People with long COVID also have higher levels of circulating S1 compared to non-PASC populations (49). The SARS-CoV-2 S protein is a surface-exposed viral receptor binding protein that can selectively trigger NLRP3 inflammasome activation and cytokine (e.g. IL-1β) secretion in macrophages derived from COVID-19 patients (48). The persistent presence of the S1 protein may cause reprogramming of monocytes/macrophages in PASC populations, allowing rapid inflammasome assembly (48).

## 4 Concerns and perspectives on the inflammatory burden from COVID-19-induced immunity trained

### 4.1 PASC

Although SARS-CoV-2 infection induced epigenetic changes in the hematopoietic system prime the host for protection from reinfection, proinflammatory monocyte phenotypes driven by epigenetic reprogramming persist even after recovery from COVID-19, representing a potential form of biological memory that could drive PASC. The pervasive effects of circulating hyperinflammatory monocytes could underpin multiorgan PASC manifestations. Some studies have already demonstrated infiltration of proinflammatory monocytes and macrophages in tissues of patients with long COVID (17, 50). One interesting study found that monocyte numbers correlated positively with the degree of CT abnormalities in the lungs of long COVID patients (50). Additionally, a recent study found that in mice infected with MHV-1 to model COVID-19, prolonged monocyte infiltration into the brain and lungs was associated with neurological symptoms and increased inflammatory microglia (17). At 30 days post-infection, monocyte numbers were increased in the brain and displayed an activated phenotype (17).

The poised inflammatory state of monocytes after COVID-19 infection can drive persistent low-grade inflammation and subsequent tissue damage, potentially contributing to PASC (17, 51). Reprogrammed monocytes exhibit increased responsiveness to stimulation even months after recovery (17), indicating the epigenetic changes prime monocytes to readily induce inflammatory cytokine production. This pre-activated state likely sustains inflammatory responses that are no longer beneficial and instead become detrimental. Prolonged release of inflammatory mediators like chemokines can have systemic effects (51) and may cause symptoms like fatigue and cognitive dysfunction as well as tissue-specific damage (52). COVID-19-associated coagulopathy may be perpetuated by monocyte-derived tissue factor production (53, 54). Additionally, monocyte recruitment and accumulation in tissues mediated by adhesion molecules and chemokines can drive localized inflammation that impairs organ function (55).

### 4.2 Possible inflammatory burden

Research has demonstrated that the number of circulating monocytes is increased in both COVID-19-recovered patients and pneumonia patients compared to the healthy group, and this increase is associated with elevated levels of inflammatory factors (56). Recent studies have found that COVID-19 can induce epigenetic changes in (HSPCs), leading to enhanced pro-inflammatory phenotypes in monocytes (17). This effect may contribute to the persistent low-grade inflammation and inflammatory tissue damage observed during the recovery process in COVID-19 patients. The infiltration and accumulation of these hyperactivated monocytes in multiple organs are associated with long COVID such as respiratory distress and cognitive impairment (17, 56, 57). This trained immune aberration induced by COVID-19 may impede inflammation resolution and delay tissue repair.

Inflammatory tendencies of the innate immune system caused by epigenetic alterations of the hematopoietic system brought about by COVID-19 may induce exacerbation of some chronic inflammatory diseases (58). A study included 105 patients with COVID-19, of whom 52 (50%) met the CDC definition of long COVID. Participants who met the definition of long-term symptoms had a median symptom duration of 193 days, compared with a median duration of 11 days for those who did not meet the definition (p<0.001). Participants reported autoimmune disease activity scores were significantly lower after COVID-19 (mean score 6.6) than before infection (mean score 7.6) (p<0.001, with higher scores indicating better self-reported disease control). 41% of respondents reported autoimmune disease flares after SARS-CoV-2 infection, with the majority of flares occurring 1-4 weeks after diagnosis. The median RAPID3 score was significantly higher in the chronically symptomatic group (11.2) than in the non-chronically symptomatic group (7.3) (p=0.0067, with higher scores indicating higher disease activity) (59). This suggests that the inflammatory response is exacerbated after COVID-19, which may be related to the inflammatory tendency of monocytes, suggesting that we need to test for the disease in relevant populations who have been infected with SARS-CoV-2 in the past and who suffer from autoimmune inflammation-related diseases (59).

The epigenetic changes in monocytes persisting after COVID-19 recovery may disrupt the resolution of inflammation needed for proper tissue repair. Efficient tissue repair requires a coordinated transition from inflammatory monocytes/macrophages to anti-inflammatory, pro-resolving macrophages (60). However, the poised inflammatory monocytes evident after COVID-19 are epigenetically programmed to sustain inflammation (17), which could impede this transition process. Thus, the monocyte reprogramming induced by COVID-19 represents an aberrant form of training that hinders the innate immune system’s capacity to resolve inflammation and facilitate tissue repair.

### 4.3 Long-term implications of trained immunity post-COVID-19

Recent findings highlight the importance of trained immunity in the aftermath of COVID-19. It appears that SARS-CoV-2 causes lasting changes in the myeloid cell population, which might affect various inflammatory conditions. This notion is exemplified by the sustained presence of T-bet+ CD16+ and IRF1+ CD14+ monocytes, which exhibit trained and activated epigenomic profiles. It seems that COVID-19’s immunological effects may linger well past the initial infection period (61). The phenomenon of trained immunity is marked by these epigenetically altered monocytes. They may play a part in shaping the inflammatory environment that fosters the progression of atherosclerosis (62). Considering atherosclerosis as an inflammatory disorder where immune dysregulation is key, the overstimulated innate immune response post-COVID-19 could potentially hasten the development of atherosclerotic conditions (63). The aggravation of inflammatory arthritis due to the trained immunity brought on by COVID-19 also raises concern. The ingrained epigenetic memory within innate immune cells and their precursors might lead to sustained inflammation, aggravating joint damage in arthritis. This issue is of particular importance since persistent immune activation within the joints is a hallmark of arthritis (64). Trained immunity may have clinical implications for post-COVID-19 respiratory issues. Abnormal immune cell populations and cytotoxic T cells in the respiratory tracts of patients with lingering respiratory symptoms post-infection point to this. Moreover, disruptions in innate immune responses may be involved in the development of multisystem inflammatory syndromes (MIS-C and MIS-A), which are characterized by severe inflammation and distinct monocyte and dendritic cell profiles (61). As research continues to uncover the breadth of long COVID’s impact, the breadth of trained immunity’s long-term effects is becoming increasingly evident. It’s imperative to delve into how these enduring immune changes affect the risk and intensity of inflammatory diseases, potentially revealing novel intervention points to alleviate these long-standing effects.

## 5 Discussion

There is a need for additional clinical studies to directly quantify inflammatory mediators and map epigenetic changes in HSPC and monocytes in COVID-19 recovered patients. These studies will help provide insight into the molecular mechanisms and dynamics of the persistent inflammatory burden resulting from epigenetic reprogramming of the hematopoietic system following COVID-19 infection. Studies directly linking monocyte epigenetic changes to specific inflammatory pathways in COVID-19 patients remain limited. Cheong et al. found increased accessibility at loci encoding cytokines like IL10, IFNG (17). While monocytes exhibit an altered inflammatory phenotype post-COVID-19, including cytokine hypersecretion (56, 65), directly attributing this to epigenetic priming requires further genome-wide investigations. Ongoing research is warranted to map COVID-19 induced monocyte epigenetic changes to functional inflammatory networks beyond individual loci. Companion efforts pairing epigenomic profiling with single cell transcriptomics and protein-level analyses in recovered patients can help validate whether chromatin alterations alter inflammatory pathway activity. Such molecular mapping may reveal new therapeutic targets within reprogrammed monocyte epigenomes to mitigate chronic inflammation post-COVID-19.

The targeting of the IL-6 signaling pathway is a promising strategy for managing chronic inflammation and trained immunity post-COVID-19. IL-6 blockade, which has been shown to reduce inflammatory gene expression, provides a basis for such treatments (17). Elevated IL-6 levels in patients recovering from COVID-19 have been associated with persistent symptoms (66), and a case report suggests that the IL-6 receptor antagonist, tocilizumab, ameliorates symptoms of PASC (67). Inhibition of IL-6 may hinder STAT3-mediated epigenetic initiation, thereby resetting the inflammatory phenotype of monocytes (17). Considering the emerging role of IL-1β in trained immunity and its exacerbating effects on post-COVID inflammation, it is imperative to explore broader research and therapeutic approaches (42–44). The synergy between elevated IL-6 and IL-1β levels in persistent symptoms post-recovery suggests their joint contribution to ongoing inflammation (42, 66). IL-1β’s involvement in the metabolic and epigenetic reprogramming of immune cells further supports this connection (43). Hence, investigating the combined inhibition of IL-6 and IL-1β could lead to more effective strategies to alleviate PASC. Future studies should focus on the IL-6 and IL-1β interaction in trained immunity, specifically their role in monocyte reprogramming and sustained inflammation. High-resolution immune monitoring and clinical trials on IL-6 and IL-1β inhibition are warranted to uncover new treatments for long COVID.

Research into the epigenetic reprogramming of monocytes and neutrophils has unveiled potential links between long-term immune changes and the risk and severity of inflammatory diseases, such as cardiovascular and joint disorders (28, 44, 56, 61–63). Two years after infection, people with novel coronavirus infections that do not require hospitalization continue to have an increased risk of coagulation and hematologic disorders, lung disease, fatigue, gastrointestinal disorders, musculoskeletal disorders and diabetes (68). Chronic inflammation may be an important cause of PASC (69). Continued research is vital to explore the association between trained immunity induced by COVID-19 and the elevated incidence or severity of inflammation-related diseases. Longitudinal monitoring of immunologic and epigenetic markers in COVID-19 recovered patients can help guide interventions to mitigate chronic inflammation (70). By quantifying cytokine levels, immune cell subpopulation frequencies, and chromatin accessibility changes in consecutive blood samples, remission or persistence of inflammation can be tracked.

An intriguing question is whether COVID-19-induced epigenetic changes are passed on to the next generation. A recent study has revealed that the trained immune phenotype induced by Candida albicans infection can be transgenerationally and transmissibly inherited in mice (71). Whether similar transgenerational epigenetic inheritance exists in humans following COVID-19 infection remains to be investigated. Whether COVID-19 causes transgenerational inheritance of epigenetic inheritance in humans is a question of interest. There is no direct evidence that COVID-19 causes transgenerational epigenetic changes in humans. Ideally, a multigenerational cohort study would answer this question, but such a study would be difficult. Understanding the potential intergenerational transmission of COVID-19-induced epigenetic changes will provide important insights into the long-term impact of the epidemic on collective human immunity.

## 6 Conclusion

In summary, the epigenetic reprogramming of monocytes in the wake of SARS-CoV-2 infection underscores a significant mechanism behind trained immunity and the enduring inflammation seen in long COVID. Reprogrammed monocytes can infiltrate tissues extensively, leading to chronic inflammation and impaired tissue repair. COVID-19 infection might worsen autoimmune diseases and influence the body’s inflammatory tendencies through epigenetic changes in the hematopoietic system. Key cytokines like IL-6 and IL-1β emerge as central players in these changes, with potential implications for tissue repair and autoimmune disease exacerbation. Further clinical studies are needed to directly assess inflammatory mediators and epigenetic changes in hematopoietic progenitor cells and monocytes in COVID-19-recovered patients. Basic research is essential to understand the mechanisms of monocyte epigenetic reprogramming and develop targeted interventions to reduce the inflammatory burden and address post-acute sequelae of SARS-CoV-2 infection. Moreover, the prospect of transgenerational transmission of these epigenetic changes calls for further investigation to understand the full impact of COVID-19 on future generations, making it a pivotal focus for ongoing research and public health initiatives.

## Data availability statement

The original contributions presented in the study are included in the article/supplementary material. Further inquiries can be directed to the corresponding author.

## Author contributions

JG: Conceptualization, Writing – original draft, Writing – review & editing. QL: Writing – original draft, Writing – review & editing. JZ: Writing – original draft, Writing – review & editing. SX: Writing – original draft, Writing – review & editing.
